# A Case of Pharyngeal Stenosis Caused by Behçet’s Disease Treated With Transoral Videolaryngoscopic Surgery

**DOI:** 10.7759/cureus.33616

**Published:** 2023-01-10

**Authors:** Yu Suekata, Kiyohito Hosokawa, Takahiro Kawasaki, Daisuke Nakatsubo, Masashi Tanida, Makoto Ogawa, Hidenori Inohara

**Affiliations:** 1 Otorhinolaryngology - Head and Neck Surgery, Osaka University Graduate School of Medicine, Osaka, JPN; 2 Respiratory Medicine and Clinical Immunology,, Osaka University Graduate School of Medicine, Osaka, JPN; 3 Respiratory Medicine and Clinical Immunology, Osaka University Graduate School of Medicine, Osaka, JPN

**Keywords:** tracheotomy, dysphagia, transoral surgery, pharyngeal stenosis, behçet’s disease

## Abstract

Behçet’s disease is a refractory inflammatory disease characterized by recurrent oral aphthous ulcers. Ulcers are commonly seen in the oral cavity and the pharyngeal region. In patients with recurrent pharyngeal ulcers, pharyngeal stenosis may occur and leads to dysphagia. Herein, we report a case of pharyngeal stenosis caused by recurrent ulcers due to incomplete Behçet’s disease. Prednisolone, colchicine, and infliximab were administered and resolved the pharyngeal ulcers, however, dysphagia persisted. To improve the swallowing function, a pharyngeal dilation surgery and transoral videolaryngoscopic surgery were performed, which resulted in an enlarged pharyngeal cavity. Oral intake of water was initiated the day after surgery, and after six days, the patient was able to take a normal diet. The pharyngeal stenosis had not recurred for one year after the surgery, and a normal diet continued without any dietary restrictions. Therefore, in a case of a severe oropharyngeal lesion, periodic follow-up and surgical interventions by an otolaryngologist are necessary.

## Introduction

Behçet’s disease is a refractory inflammatory disease characterized by recurrent oral and genital aphthous ulcers, uveitis, and skin lesions [1,2]. In particular, oral aphthous ulcers are the most frequent manifestation, seen in all patients at some time in the clinical course [2]. Aphthous ulcers often occur in the oral cavity and the pharyngeal region, characterized by scarring during healing [3]. Scar contracture may result in pharyngeal stenosis, although severe stenosis that limits swallowing is rare [3,4]. Among the surgical treatments for this condition [4-9], flap reconstruction or an expansion procedure is recommended [4,5,7,8]. Herein, we report a rare case of pharyngeal stenosis due to recurrent pharyngeal ulceration caused by incomplete Behçet’s disease that was successfully treated by transoral videolaryngoscopic surgery (TOVS).

## Case presentation

**Figure 1:**
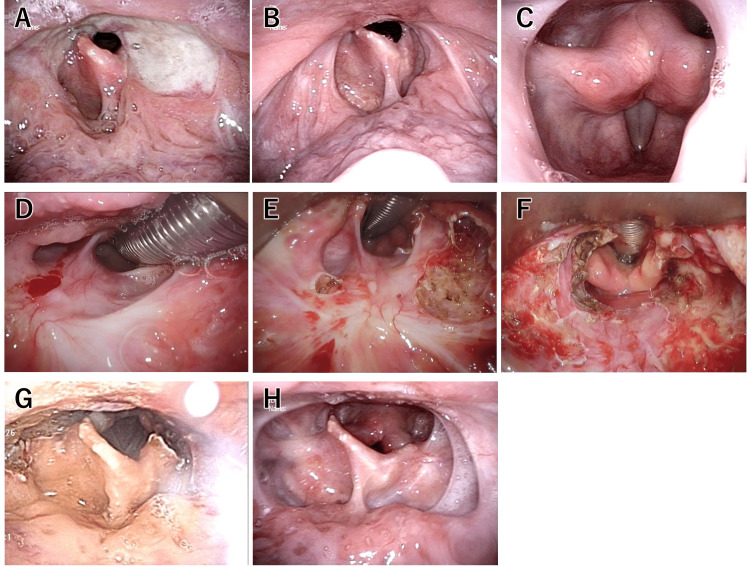
Pharyngeal findings of the case. (A) Pharyngeal ulcer and stenosis before treatment. (B, C) The pharynx and larynx after administration of infliximab. An ulcer is completely resolved, but stenosis progresses. During the surgery after exposure using FK-WO retractor (D), partial resection of stenosis (E), and completion of the expansion of the pharynx (F). The pharynx two days (G) and one year (H) after the surgery.

A 24-year-old male was referred to our hospital due to progressively worsening dysphagia. His history included multiple oral aphthae for 11 years of age. Incomplete Behçet’s disease was diagnosed due to recurrent oral aphthous ulcers, genital ulcers, and folliculitis/erythema nodosum. The patient was followed up at the department of rheumatology and otorhinolaryngology in our hospital. Since his condition appeared to be stable on oral prednisolone 5 mg and colchicine 0.5 mg, otolaryngological follow-up was stopped for six years. However, he was hospitalized due to acute epiglottitis presenting with ulcer and pharyngeal stenosis, which was treated with steroids and antibiotics. Two weeks later, he was referred to our otorhinolaryngology department for close examination and treatment of residual pharyngeal stenosis and ulcerative lesions. Video laryngoscopy revealed a circumferential oropharyngeal stenosis with a large ulcer at the level of the epiglottis (Figure 1A). A flare-up of Behçet’s disease was suspected to cause the pharyngeal ulcer and stenosis, rheumatologists first controlled the flare-up before performing the surgical treatment.

On physical examination, none of the following were observed: ocular conjunctival abnormalities, temporal artery tenderness, palpable cervical lymph nodes, enlarged thyroid gland, cardiopulmonary murmur, abdominal abnormalities, normal extremity joints, lower extremity edema, skin rash or induration, nor central or peripheral abnormalities. No abnormalities were detected on upper and lower gastrointestinal endoscopy, nor signs suggestive of active uveitis or Behçet’s disease on ophthalmologic examination, nor neurogenic Behçet’s disease on head MRI, nor vascular lesions suggestive of vascular Behçet’s disease on contrast-enhanced CT scan (head, neck, chest, and abdomen). A biopsy of the pharyngeal stenosis showed inflammation without findings suggestive of cancer. Thus, it was determined that the pharyngeal ulcer was due to a flare-up of incomplete Behçet's disease, which had been diagnosed 13 years earlier. The dose of prednisolone was increased to 30 mg while maintaining colchicine 1 mg and starting infliximab at 5 mg/kg. After the third dose of infliximab, the disease seemed controlled based on the resolved sore throat for the first time since the onset of incomplete Behçet’s disease and the disappearance of the pharyngeal ulcer on laryngoscopy (Figures 1B, 1C).

Prednisolone was gradually tapered to 10 mg before surgery. Two months after the first dose of infliximab, pharyngeal dilation surgery with TOVS was performed. During oral tracheal intubation, a spiral tube (6.0 mm inner diameter) with a bronchial fiberscope was inserted into the glottis-trachea. To dilate the pharynx, the intubation tube was evacuated forward using an FK-WO retractor, but the supraglottic area was not visible due to stenosis (Figure 1D). An electric needle knife (KD-600, Olympus, Tokyo) was used to incise the thin mucosa at the stenosis, which spontaneously opened by tension using the FK-WO retractor (Figure 1E). During the left lateral incision, a branch of the superior laryngeal artery suddenly bled, which was easily ligated with a hemostatic clip. To prevent the recurrence of stenosis, triamcinolone acetonide was injected at the incision site. Finally, we confirmed the pharyngeal cavity had been enlarged (Figure 1F) and terminated the surgery. Operative time was 102 min, with minimal blood loss.

Postoperative laryngeal edema was mild (Figure 1G). Oral intake was started the next day, and normal diet was consumed from the sixth postoperative day. The patient was finally discharged on the seventh postoperative day. One year postoperatively, restenosis of the pharynx did not occur (Figure 1H), and the patient was able to have a normal diet without any dietary restrictions. The overall activity of Behçet’s disease is well controlled with prednisolone 2 mg, colchicine 1 mg, and infliximab 5 mg/kg every eight weeks.

## Discussion

Pharyngeal stenosis is caused by epithelial or mesenchymal neoplasms [10] as well as scar formation during the healing process of various inflammatory and traumatic injuries to the pharynx, including infections, dermatological and medical systemic diseases, autoimmune diseases, tumors, and surgical procedures. Repeated ulceration due to these causes results in scarring and stenosis. Behçet’s disease is one of the diseases that cause pharyngeal stenosis due to recurrent pharyngeal ulcerative lesions.

Behçet’s disease is a disease characterized by recurrent inflammation of unknown origin in various organs throughout the body and mainly presents with oral aphthous ulcers, genital ulcers, uveitis, and skin lesions [1,2]. Secondary symptoms include arthritis, epididymitis, gastrointestinal lesions, vascular lesions, and central nervous system lesions. For oral aphthous ulcers and pharyngeal ulcers, as in this case, topical or systemic steroids and colchicine are recommended. TNF-α inhibitors such as infliximab are also effective and are considered Recommendation A with Level of Evidence 1B in practice guidelines [1]. In the present case, pharyngeal stenosis was controlled by early surgical treatment because the pharyngeal ulcer healed very rapidly with the increased dose of prednisolone and the addition of infliximab.

Several treatments are possible for pharyngeal stenosis in cases of Behçet’s disease and similar refractory pharyngeal ulcers [4-9]. Effective approaches are to enlarge the narrowed area by excision of the scar, transplant the mucosa or skin to the fresh surface area, and reconstruct with a pedicle or free flap [5-8]; transpire and excise thickened mucosa by electrocautery or laser ablation [9]; and perform endoscopic dilatation [6]. Given the variety of techniques, treatment selection should be based on the location and degree of stenosis.

In the present case, although the pharyngeal stenosis was circumferential, it was confined to the level of the epiglottis and there was no stenosis in the hypopharynx or larynx. Therefore, we investigated the possibility of minimally invasive surgery. In 2005, Fujimaki et al. reported a case of pharyngeal stenosis similar to the present case in which the pharyngeal stenosis was controlled by transoral surgery using a direct laryngoscope with tracheostomy 9). In this case, we believed that an oral procedure that did not require a tracheostomy would be best for the patient. In a preoperative conference with our anesthesiologists, the consensus was reached to first attempt intubation with endoscopic assistance, and if intubation was unsuccessful, a tracheostomy would be performed. As a result, the intubation was successful and the pharyngeal dilation was sufficiently achieved, which did not require a tracheostomy.

The FK-WO retractor used to expand the pharynx in this surgery is mainly used for TOVS of pharyngeal malignancies. However, in our department, TOVS is also performed for benign pharyngeal lesions such as laryngeal papilloma, pharyngeal vascular malformation, and lateral fixation of the vocal folds. In the present case, the pharynx was widely dilated using the FK-WO retractor, and a wide-angle operative field was obtained by a rigid endoscope with a freely curved tip. As the FK-WO retractor also applies strong tension to the pharynx, the degree of tension release at the incision site can be observed during surgery. In the pharyngeal region, the superior laryngeal artery and its branches are the main source of bleeding, which can be difficult to stop without hemostatic devices. In the present case, using an FK-WO retractor allowed for a wider operative field and easy use of hemostatic devices.

In the present case, the diagnosis of incomplete Behçet’s disease was made before the appearance of pharyngeal stenosis. However, since the patient did not appear in his otorhinolaryngological follow-ups for 6 years, the stenosis was detected when it was highly advanced, leading to the development of dysphagia. Had the pharyngeal stenosis been detected early, intubation for general anesthesia might have been easier. Thus, otolaryngologists are recommended to participate in the management of Behçet’s disease [5]. Therefore, in the case of a severe complication of a severe oropharyngeal lesion, periodic follow-up by an otolaryngologist is absolutely necessary.

## Conclusions

We experienced a case of pharyngeal stenosis caused by incomplete Behçet’s disease. A high-dose prednisolone and addition of infliximab result in the complete disappearance of the pharyngeal ulcer in a short period of time, allowing prompt and effective surgical treatment, such as TOVS, for pharyngeal stenosis. Long-term follow-up is recommended for oropharyngeal ulcers associated with Behçet’s disease because of the future risk of stenosis.
